# ZnJ2 Is a Member of a Large Chaperone Family in the Chloroplast of Photosynthetic Organisms that Features a DnaJ-Like Zn-Finger Domain

**DOI:** 10.3389/fmolb.2018.00002

**Published:** 2018-02-15

**Authors:** Lior Doron, Pierre Goloubinoff, Michal Shapira

**Affiliations:** ^1^Department of Life Sciences, Ben-Gurion University of the Negev, Beer Sheva, Israel; ^2^Department of Plant Molecular Biology, Faculty of Biology and Medicine, University of Lausanne, Lausanne, Switzerland

**Keywords:** ZnJ2, DnaJ-like chaperone, RNaseA assay, redox, chloroplast chaperones

## Abstract

Photosynthesis is performed by large complexes, composed of subunits encoded by the nuclear and chloroplast genomes. Assembly is assisted by general and target-specific chaperones, but their mode of action is yet unclear. We formerly showed that ZnJ2 is an algal chaperone resembling BSD2 from land plants. In algae, it co-migrates with the *rbcL* transcript on chloroplast polysomes, suggesting it contributes to the *de-novo* synthesis of RbcL (Doron et al., 2014). ZnJ2 contains four CXXCXGXG motifs, comprising a canonical domain typical also of DnaJ-type I (DNAJA). It contributes to the binding of protein substrates to DnaK and promotes an independent oxidoreductase activity (Mattoo et al., 2014). To examine whether ZnJ2 has oxidoreductase activity, we used the RNaseA assay, which measures the oxidation-dependent reactivation of reduced-denatured RNaseA. Although ZnJ2 assisted the native refolding of reduced-denatured RNaseA, its activity was restricted to an oxidizing environment. Thus, ZnJ2 did not carry the exclusive responsibility for the formation of disulfide bridges, but contributed to the stabilization of its target polypeptides, until they reached their native state. A ZnJ2 cysteine deficient mutant maintained a similar holding chaperone activity as the wild-type and did not induce the formation of disulfide bonds. ZnJ2 is devoid of a J-domain. It thus does not belong to the J-domain co-chaperones that target protein substrates to DnaK. As expected, *in vitro*, its aggregation-prevention activity was not synergic to the ATP-fueled action of DnaK/DnaJ/GrpE in assisting the native refolding of denatured malate dehydrogenase, nor did it show an independent refolding activity. A phylogenetic analysis showed that ZnJ2 and BSD2 from land plants, are two different proteins belonging to a larger group containing a cysteine-rich domain, that also includes the DNAJAs. Members of this family are apparently involved in specific assembly of photosynthetic complexes in the chloroplast.

## Introduction

Molecular chaperones are intimately involved in the synthesis of nascent polypeptides, their proper folding and hence their subsequent assembly into complexes that comprise multiple subunits. The DnaK/DnaJ/GrpE chaperone system is a canonical Hsp70/Hsp40 network that is conserved during evolution, whereby bacterial Hsp70, DnaK, possesses an energy-driven active unfolding capacity (Sharma et al., 2010). In contrast to DnaK, DnaJ cannot hydrolyze ATP, but can target specific substrates to active refolding by DnaK (Mccarty et al., 1995; Martin and Hartl, 1997). DnaJ can also function as an independent chaperone, mainly by holding and stabilizing the unfolded polypeptide substrates through protein-protein interactions (Szabo et al., 1996; Lu and Cyr, 1998).

The tertiary structure of proteins is often stabilized by disulfide bridges, formed by polypeptides that contain cysteine residues. Disulfide bonds are generated *in vivo* by thiol-disulfide oxidoreductases, which can be promoted by proteins that relate to different families. One such family of proteins is the thioredoxin family that is defined by the presence of two vicinal CXXC motifs and a typical tertiary structure known as the thioredoxin fold (Holmgren, 1985; Ellgaard and Ruddock, 2005). Thioredoxins can reduce thiol groups of target proteins through a conserved CPGC element, allowing for their re-oxidation by thioredoxin oxidases, in a cascade manner (Berndt et al., 2008; Mavridou et al., 2011). These dual functions can be performed by a single protein, such as the protein di-isomerase (PDI, Carvalho et al., 2006; Appenzeller-Herzog and Ellgaard, 2008). The second family of proteins that have oxidoreductase activity are the DnaJ-like proteins. Apart from the DnaK-dependent activity of DnaJ as a co-chaperone, or its independent chaperone activity, DnaJ can also function as a reductase through its cysteine-rich domain, which in eukaryotes is present in all DnaJ type I (DNAJAs) but not in DnaJ type II (DNAJBs). The formation and reduction of disulfide bonds via its CXXCXGXG motif, is shown by the effect of DnaJ on the oxidative refolding of reduced and denatured RNaseA (rdRNaseA) *in vitro* (Tang and Wang, 2001; Shi et al., 2005; Mattoo et al., 2014). This type of activity was observed despite the fact that DnaJ does not possess a typical thioredoxin fold. Interestingly, cysteines from this domain have been shown to contribute to the “holding” activity of DNAJ (Linke et al., 2003).

Chloroplast complexes comprise of multiple subunits, some are encoded by the chloroplast genome, others by the nucleus. The latter are synthesized in the cytoplasm and are transported into the chloroplast for assembly into the mature complexes. RuBisCO is an example for a complex that comprises eight large subunits (RbcL) encoded by the chloroplast genome, and eight small subunits (RbcS) encoded by a family of nuclear genes (Gutteridge and Gatenby, 1995). RuBisCO synthesis and assembly is promoted by a group of auxiliary proteins or chaperones, whose function is currently being investigated (Brutnell et al., 1999; Wostrikoff and Stern, 2007; Liu et al., 2010; Feiz et al., 2012, 2014). One of these proteins was identified in maize, as the Bundle Sheath Defective Gene 2 (BSD2, Brutnell et al., 1999), and further investigated later on in tobacco (Wostrikoff and Stern, 2007).

We previously reported on a *Chlamydomonas reinhardtii* ortholog of BSD2, denoted ZnJ2. It is a small protein involved in the early stages of the RbcL synthesis in *C. reinhardtii*, essentially during translation elongation (Doron et al., 2014). ZnJ2 contains a conserved cysteine-rich domain (CXXCXGXG), a specific subdomain that distinguishes DnaJ type I (DNAJAs) from DnaJ type II (DNAJBs, classification according to Kampinga et al., 2009), both being J-domain containing co-chaperones that target substrate proteins for processing by DnaK. However, despite its bioinformatics annotation as a DnaJ-like protein, ZnJ2 does not contain the conserved J-domain that is responsible for the interaction between DnaJ and DnaK, and is therefore not considered as a canonical member of the DnaJ family. Previously we showed that ZnJ2 comigrates with the *rbcL* transcript on polysomes. Under normal conditions both are found in the heavier fractions, and when translation of RbcL stops in response to oxidative stress, both ZnJ2 and the *rbcL* transcript accumulate in lighter fractions that contain the 80S ribosomes. We further established that ZnJ2 has a “holding” chaperone activity, since it prevented the aggregation of denatured citrate synthase, in a typical chaperone assay (Doron et al., 2014). However, ZnJ2 did not possess reductase activity and could not reduce disulfide bridges in the insulin assay, as demonstrated for the bacterial DnaJ (Tang and Wang, 2001). Instead, once these were reduced by DTT, ZnJ2 could bind the free thiol groups and further function as a chaperone that prevented the aggregation of its β-chains. This highlighted the difference between ZnJ2 and DnaJ.

Here we show that ZnJ2 binds zinc ions through a conserved cysteine-rich center comprised of four CXXCXGXG motifs, which together form a Zn-binding domain that resembles that found in DnaJ type I. We further exclude the possibility that ZnJ2 can induce the formation of disulfide bridges by oxidation, using the RNase A refolding assay. However, despite its inability to function as an oxidoreductase under reducing conditions, we show that ZnJ2 functions as a potent chaperone that stabilizes the refolded structure of RNase A. The subsequent formation of the mature disulfide bridges may therefore occur only in an oxidizing environment.

We further highlight here another difference between the chaperone function of ZnJ2 and DnaJ, by using the malate dehydrogenase refolding assay (Horwitz et al., 1998). Unlike DnaJ, ZnJ2 does not assist the refolding activity of DnaK *in vitro* and its activity is restricted to “holding” its substrate, therefore supporting the possibility that ZnJ2 functions as a “holding” chaperone of nascent chains during translation elongation. Based on its ability to also bind free thiol groups in the insulin assay, we propose that ZnJ2 can hold onto the nascent RbcL chains and prevent the formation of premature and non-native disulfide bridges.

Finally, although ZnJ2 shares a functional domain with DnaJ Type I (Kampinga and Craig, 2010), a phylogenetic analysis shows that the chloroplast contains a multitude of such proteins, all characterized by the presence of a CXXCXGXG- based domain, and that ZnJ2 forms an independent clade in the analysis of these chaperones, which are involved in the synthesis and assembly of chloroplast complexes, the most abundant of which being RuBisCO. Our findings further emphasize the complexity and tight regulation required for the generation of photosynthetic complexes within the chloroplast.

## Materials and methods

### ZnJ2 purification

The coding sequences of ZnJ2 (lacking the first 39 amino acids) or of the Cys → Ser mutant were cloned in-frame into the pET30-GBFusion1 expression vector downstream of a T7 promoter and in-frame with C-terminal His-tag. A bacterial culture (10 ml) in LB medium supplemented with 100 μg/ml kanamycin was inoculated with a single colony of *E. coli* strain BL21 and were grown overnight at 37°C. After 12 h growth 1 ml of bacterial culture were transferred to 1 L of LB medium, supplemented with 100 μg/ml kanamycin, and allowed to grow for further 4 h. Expression of the His-tagged pET-30 GB1-ZnJ2 was induced upon addition of 0.5 mM IPTG to 1L of bacterial culture (OD_600_ = 0.5–0.7) at 20°C for 16 h. The culture was harvested, washed with 20 mM Tris-HCl, pH 8.0, and resuspended in buffer A (20 mM Tris-HCl, pH 8.0, 300 mM NaCl, 20 mM imidazole) containing 0.02% Triton X-100 and a protease inhibitor cocktail and 5 μg/ml DNaseI. The cells were disrupted in a French Press at 1500 psi and centrifuged at 45,000 rpm (Beckman 70 Ti rotor) and the sup was loaded on a HisTrap HP Ni-NTA column (5 mL, GE Healthcare) equilibrated in buffer A and purified on AKTA prime plus. After washing the column with 7.5 column volume (CV) ml of buffer A, ZnJ2 was eluted with a linear gradient from 20 to 500 mM imidazole in buffer A. Fractions of 5 mL were collected and analyzed on 15% SDS-PAGE. Zn2 was found in the fractions that contained between 100 and 200 mM Imidazole and these were combined and dialyzed (X100, dialysis membrane Tubes, MWCO 10K Cellu-Sep) overnight against 2L of buffer B (20 mM Tris-HCl, pH 8.0, 40 mM NaCl, 4 mM DTT). As a next purification step, the protein was loaded over a HiTrap Q-Sepharose column (1 mL, GE Healthcare) equilibrated in buffer B. After washing the column with 10 CV mL of buffer B, ZnJ2 was eluted between 300 and 400 mM NaCl with a linear gradient from 40 to 1000 mM NaCl in buffer B. The eluted ZnJ2 was concentrated by size exclusion ultrafiltration membrane (Amicon Ultra 15 ml, MWCO 3K) and dialyzed overnight at 4°C against buffer C (40 mM Tris-HCl, pH 8.0, 100 mM NaCl). Protein concentration was determined by absorption spectroscopy (ε280 = 14,440 M^−1^cm^−1^).

### The PAR-PCMB Zn-binding assay

Zn binding by ZnJ2 was determined using the PAR-PCMB assay, as described in Hunt et al. (1985), except that thiol bound zinc was released with para-chloromercuribenzoic acid (PCMB). Zinc release was measured by its interaction with 4-(2-Pyridylazo)resorcinol (PAR) at 500 nm, and compared to a ZnCl_2_ standard curve. Metal-free buffers were used throughout the assay, following treatment with Chelax 100 resin (5 gr in KH2PO4 buffer), for 1 h at 37°C. ZnJ2 (3 μM) was mixed with 0.1 mM PAR in 40 mM KH_2_PO_4_ buffer to measure any free or loosely bound zinc in the solution. Addition of 30 μM PCMB to the protein solution caused immediate zinc release and allowed the determination and calculation of the total amount of bound zinc per ZnJ2 molecule. PAR in buffer KH_2_PO_4_ was used as blank.

### Reduction and denaturation of RNaseA

Reduced and denatured RNaseA (rdRNaseA) was prepared as previously described by overnight incubation of the native enzyme (20 mg/ml) in 500 μl of 0.1 M Tris-HCl pH 8.6, containing 150 mM DTT and 6M guanidinium hydrochloride. Excess DTT and guanidinium hydrochloride were separated from the rdRNaseA using a Sephadex G-25 buffer replacement column equilibrated with 10 mM HCl. RNaseA aliquots (10 mg/ml stock) were stored at −80°C.

### Reactivation of reduced and denatured RNaseA

Refolding of rdRNaseA was initiated by 200-fold dilution of the protein (to a final concentration of 50 μg/mL (3.8 μM) in 1 mL of reactivation buffer (0.1 M Tris-HCl pH 7.0, 0.1 M NaCl and 1 mM EDTA). The refolding was performed in the presence or absence of ZnJ2, at changing concentrations (5, 10, and 30 μM). Aliquots (50 μL) were removed at various intervals and mixed with 50 μL of the assay mixture containing 0.1 M Tris- HCl pH 7.2, 0.1 M NaCl and 0.3 mg/ml cytidine 2′:3′-cyclic monophosphate. RNaseA activity was measured by monitoring the hydrolysis of cytidine 2′:3′-cyclic monophosphate at 284 nm. The hydrolysis was calculated as the difference between OD_284_ at *t* = 0 min and *t* = 10 min. Refolding was presented as percentage hydrolysis of treated samples, compared to the hydrolysis of native RNaseA.

To measure the effect of the oxidizing environment on rdRNaseA refolding, the assay was performed in the presence of oxidized glutathione (GSSG). rdRNaseA was renatured as described above, but the reactivation buffer contained either different concentrations of GSSG or a mixture of oxidized and reduced glutathione (GSSG:GSH 50 μM:500 μM). Assays performed under complete anaerobic conditions were done in a BD GazPak EZ pouch system that provides an anaerobic environment, during 3 days, in the presence or absence of ZnJ2 (5 and 10 μM).

### Malate dehydrogenase refolding assay

Native malate dehydrogenase (MDH, Sigma, at a final concentration of 0.7 μM) was heat- denatured during 30 min at 47°C, in 100 μL of a buffer containing 50 mM Tris-HCl pH 7.5, 20 mM MgAc_2_, 150 mM KCl, and 5 mM dithiothreitol (DTT). The denaturation took place in the absence, or presence of different chaperone combinations. These included DnaK (6 μM)/ATP (4 mM) and DnaJ (1 μM), DnaK/ATP and ZnJ2 (1 μM), DnaK/ATP along with DnaJ and ZnJ2, DnaK/ATP, DnaJ alone and ZnJ2 alone. GrpE (1 μM) was added to the assay tubes that contained DnaK/ATP.

Refolding was monitored at 25°C in the presence of 3 mM phosphoenolpyruvate and 20 μg/mL pyruvate kinase to regenerate ATP. Aliquots were removed at various intervals (0, 10, 40, 80, and 160 min) and MDH activity was monitored at 340 nm, during 40 s. The percentage of refolding was calculated relative to the initial 0.7 μM native MDH activity before denaturation.

### Multiple sequence alignment and generation of a phylogenetic tree

The amino acid sequences of ZnJ2-orthologs from representative organisms were obtained from Phytozome v10.1 (http://phytozome.jgi.doe.gov/pz/portal.html) by a eukaryotic restricted BLAST search based on the amino acid sequence of the algal BSD2-ortholog, ZnJ2 from *Chlamydomonas reinhardtii* (Cre03.g201050.t1.2) and maize BSD2 (GRMZM2G062788_T01). The sequences were introduced into the ClustalW2–Multiple Sequence Alignment EBI server, (http://www.ebi.ac.uk/Tools/services/web/toolform.ebi?tool=clustalw2). The pairwise alignment (slow) was generated by the ID Protein Weight Matrix and the multiple sequence alignment was generated by the Gonnet Protein Weight Matrix (Larkin et al., 2007; Goujon et al., 2010). The retrieved sequences were then subjected to ClustalW2 multiple sequence alignment to generate a neighbor-joining phylogenetic tree (Clustal W2_Phylogeny) by the bootstrap test (Saitou and Nei, 1987).

## Results

### ZnJ2 is a Zn binding protein

**Figure 1:**
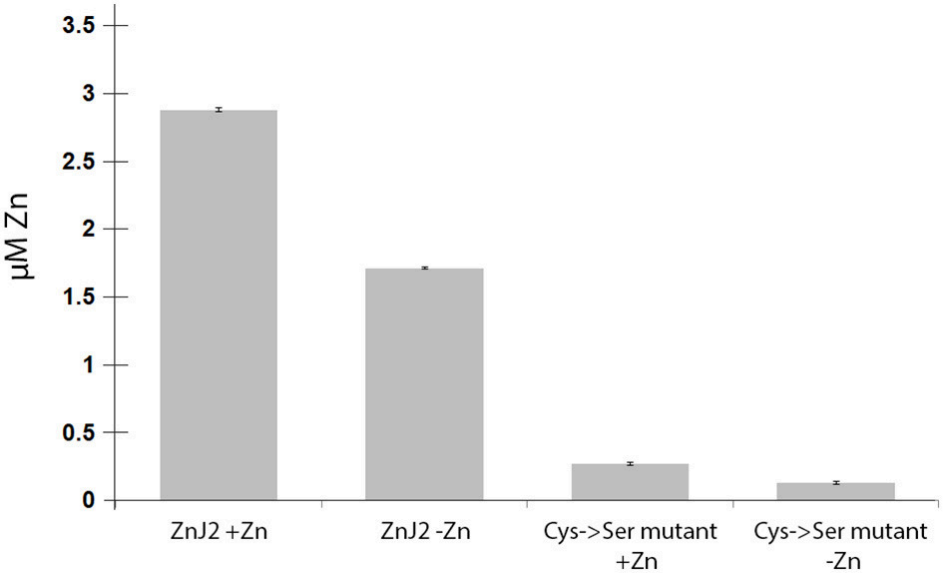
ZnJ2 is a Zinc-binding protein. Purified recombinant ZnJ2 (3 μM) or its Cys->Ser mutant were pre-incubated with 100 μM ZnCl2 to obtain full saturation of the tested proteins with bound zinc (+Zn). Control samples of both proteins were not treated with zinc before the assay (–Zn). The proteins were then dialyzed against free-metal buffer and further incubated in 1 mL buffer containing 0.1 mM PAR in 40 mM KH_2_PO_4_. The zinc release was obtained using PCMB and the PAR(Zn)_2_ complex was monitored at 500 nm. Each assay was performed in triplicates.

The partial similarity of ZnJ2 with DnaJ, especially in the CXXCXGXG motif, suggests that ZnJ2 can bind Zn ions through its cysteine-rich domain. The purified recombinant ZnJ2 from *C. reinhardtii* was pre-incubated with 100 μM ZnCl2 and 4 mM DTT, to saturate the protein with Zn. Zn was then released with PCMB and further allowed to interact with PAR. The addition of 30 μM PCMB caused the release of one zinc atom per protein molecule (the addition of a higher concentration of PCMB did not release additional zinc, data not shown). The recombinant ZnJ2 released Zinc even if it was not pre-saturated with zinc prior to the addition of PCMB, although this release was somewhat lower, indicating that the recombinant protein was partially loaded with endogenous zinc. The requirement of the cysteine-rich center for the zinc-binding was further verified using a mutant ZnJ2 in which all the cysteines were replaced by serine residues (Cys->Ser). No zinc release was observed in the Cys->Ser mutant, even after the protein was pre-incubated with zinc before the assay. This suggested that the cysteine-rich region is required for the zinc-binding of ZnJ2 (Figure 1).

### ZnJ2 promotes the oxidative refolding of RNaseA

**Figure 2:**
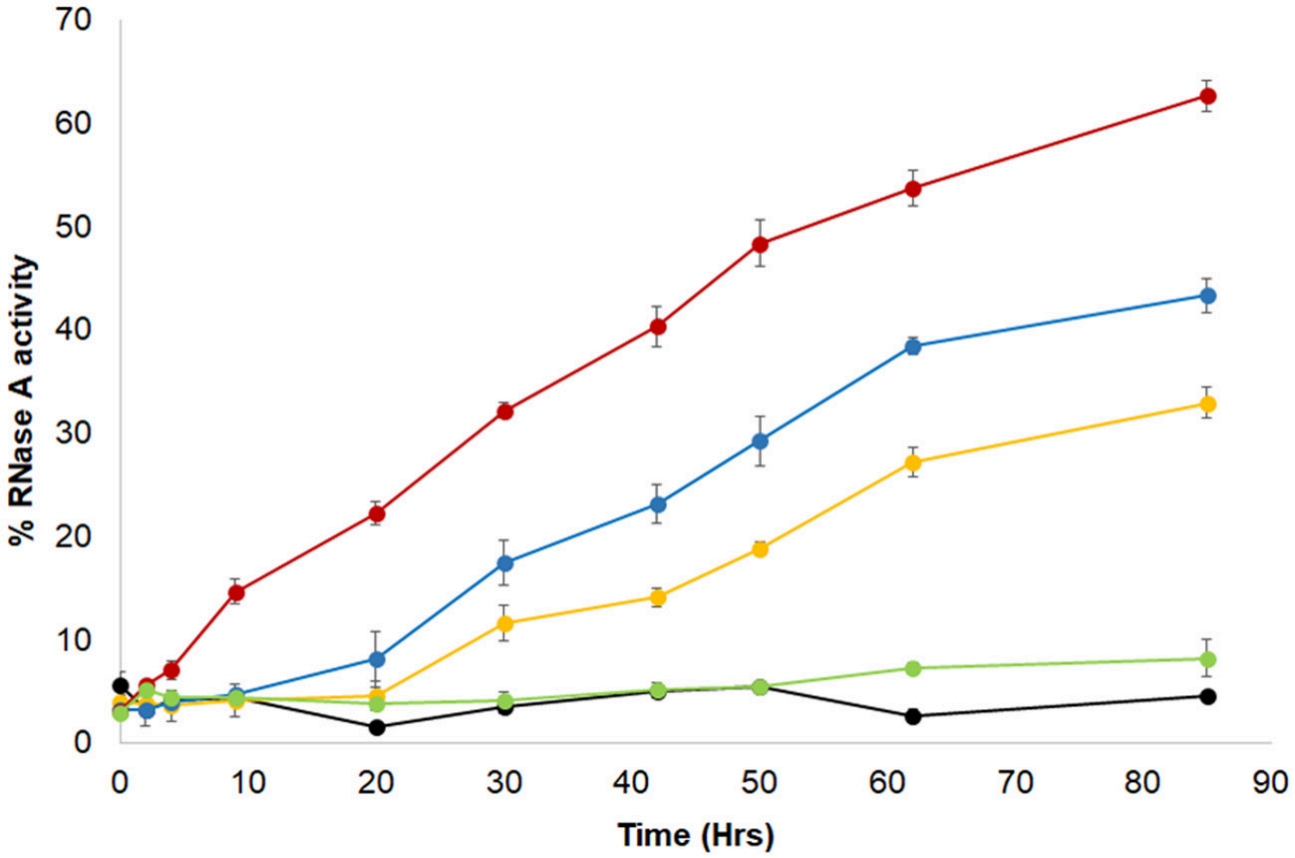
ZnJ2 affects the oxidative refolding of reduced and denatured RNaseA. RNaseA (10 mg/ml) was denatured in the presence of 150 mM DTT and 6 M guanidine hydrochloride. Refolding of the reduced and denatured RNaseA was initiated by its dilution (200-fold) into renaturation buffer to a final concentration of 50 μg/mL (3.8 μM). Refolding was either spontaneous (•) or conducted in the presence of 5 (•), 10 (•), or 30 μM (•) ZnJ2 or 5 μM citrate synthase as a negative control that replaced ZnJ2 (•), that served as a negative control. Aliquots were removed at various intervals and transferred into the assay mixture. RNaseA activity was measured by monitoring the hydrolysis of cytidine 2′:3′-cyclic monophosphate at 284 nm. The percentage of refolding was calculated relative to native RNaseA activity. Similar results were obtained in three independent replicate experiments.

The algal BSD2-ortholog, ZnJ2, contains a cysteine-rich domain comprised of four CXXCXGXG elements, which are common to members of the DNAJA protein subfamily. Its earlier observed ability to protect artificially unfolded protein substrates from aggregation, led us here to examine whether ZnJ2 may also assist the formation of disulfide bridges in the substrate by an RNaseA refolding assay (Pigiet and Schuster, 1986). The native structure of RNaseA is stabilized by several disulfide bridges. Once these bonds are reduced and the protein denatured, it loses its native structure and activity. Recovery of RNaseA enzymatic activity requires that the protein refolds into its native structure, but this fold must further be stabilized by the formation of disulfide bonds (Anfinsen et al., 1961; Haber and Anfinsen, 1961). Earlier studies showed that bacterial DnaJ is capable of catalyzing the refolding of reduced and denatured RNaseA (rdRNaseA). It contributes to the oxidative refolding of rdRNaseA by assisting the reformation of native disulfide bridges *in vitro* (Tang and Wang, 2001). To establish whether ZnJ2 could have a similar activity, the oxidative refolding of rdRNaseA was measured in the presence of recombinant ZnJ2. However, unlike bacterial DnaJ, which was shown to readily carry out refolding of rdRNaseA within a few minutes, our addition of ZnJ2 could assist the refolding of rdRNaseA only after a prolonged incubation of up to 80 h (Figure 2, red circles). Yet, this happened in a dose-dependent manner that was based on the molar ratio between ZnJ2 and the substrate (light blue and orange circles). Decreasing this ratio reduced the amount of refolded and active RNaseA, and no refolding was observed when ZnJ2 was replaced by a non-related protein, such as citrate synthase (light green circles), or in a control assay that lacked ZnJ2 (black circles). Thus, as demonstrated by the slow reappearance of RNaseA activity, these findings indicate that although ZnJ2 could assist the refolding of RNaseA, its function was very slow, suggesting that it did not resemble that of DnaJ.

### ZnJ2 is a chaperone that functions in a thiol independent manner *in vitro*

**Figure 3:**
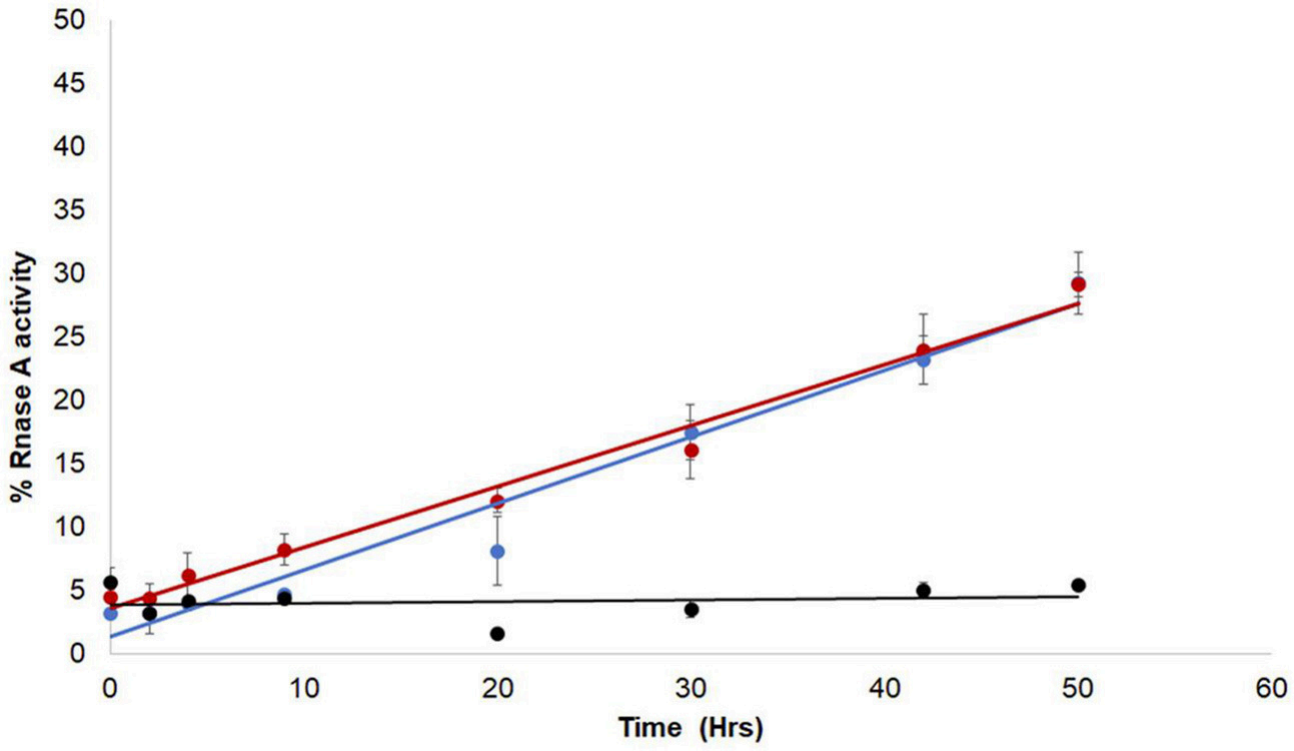
The Cys-deficient mutant form of ZnJ2 is capable of oxidative refolding of reduced and denatured RNaseA. RNaseA was reduced and denatured as described in Figure 2. Refolding of the reduced and denatured RNaseA was initiated by its dilution (200-fold) into renaturation buffer to a final concentration of 50 μg/mL. Refolding was either spontaneous (•) or conducted in the presence of 10 μM WT ZnJ2 (•), or 10 μM ZnJ2 Cys->Ser mutant (•). Aliquots were removed at various intervals and transferred into the assay mixture. RNaseA activity was measured by monitoring the hydrolysis of cytidine 2′:3′-cyclic monophosphate at 284 nm. The percentage of refolding was calculated relative to native RNaseA activity. Similar results were obtained in three independent replicate experiments.

Since the enzymatic activity of RNaseA depends on the formation of native disulfide bonds in the folded polypeptide, the role of the ZnJ2 thiol groups was examined by generating a mutant ZnJ2 in which all the cysteines were replaced by serine residues (Cys->Ser), and testing its ability to assist the refolding of RNaseA. Figure 3 shows that the ZnJ2 mutant was able to restore the RNaseA activity as the wild-type protein (red and blue circles, respectively), suggesting that ZnJ2 does not function as an oxidase.

**Figure 4:**
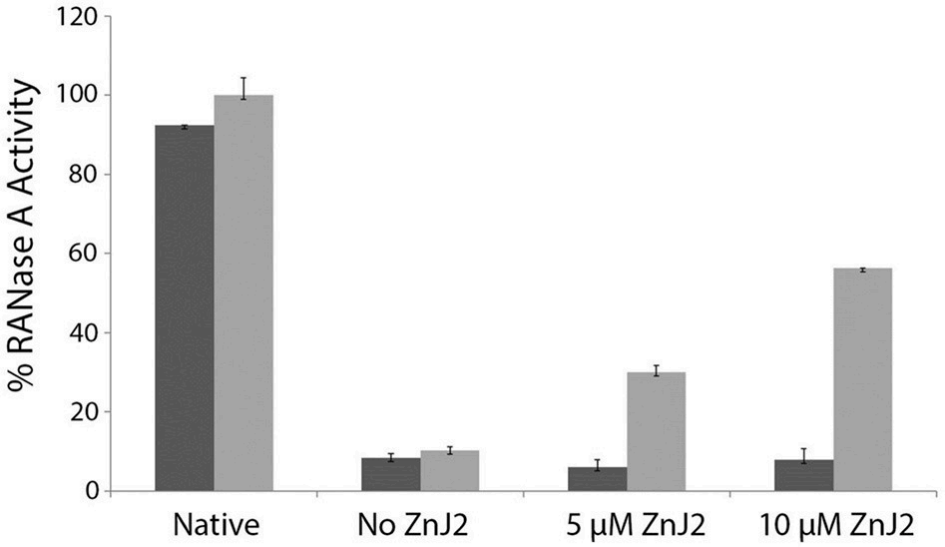
ZnJ2 cannot promote refolding of reduced-denatured RNaseA in an anaerobic environment. RNaseA was reduced and denatured as described in Figure 2. Refolding of reduced and denatured RNaseA was initiated by its dilution (200-fold) into renaturation buffer to a final concentration of 50 μg/mL. Refolding was either spontaneous (marked as No ZnJ2) or performed in the presence of 5 μM ZnJ2 or 10 μM ZnJ2 in the presence (light gray bars) or absence (dark gray bars) of air oxygen. Aliquots were removed after 80 h and transferred into the assay mixture. RNaseA activity was measured by monitoring the hydrolysis of cytidine 2′:3′-cyclic monophosphate at 284 nm. The percentage of refolding was calculated relative to native RNaseA activity (Native). Bars represent standard deviations of three readings in the assay. The experiment was repeated twice, and similar results were obtained.

Since ZnJ2 was excluded to function as an oxidase, it remained unclear how the disulfide bridges of RNaseA were reconstituted, by either the wild-type or the cysteine-less mutant of the protein. One possibility was that oxygen from the air promoted this activity. As expected, it should occur slowly and necessitating a very long period of incubation time (30–80 h) required for refolding of RNaseA, as shown in Figure 2. To verify that the reoxidation of the disulfide bridges was indeed promoted by the O_2_ from the air, refolding of rdRNaseA was examined in a dedicated anaerobic chamber, with and without the presence of ZnJ2. Results shown in Figure 4 show that in a mild oxidizing environment such as air, when re-oxidation was rather slow, the refolding was observed (up to 60% of the original activity) only in the presence of ZnJ2 (10 μM ZnJ2). However, under anaerobic conditions, no RNaseA reactivation was observed with or without ZnJ2, confirming that, ZnJ2 needed O_2_ to promote the oxidative renaturation of rdRNaseA.

### Rapid oxidation of rdRNaseA does not require ZnJ2 for its reactivation *in vitro*

**Figure 5:**
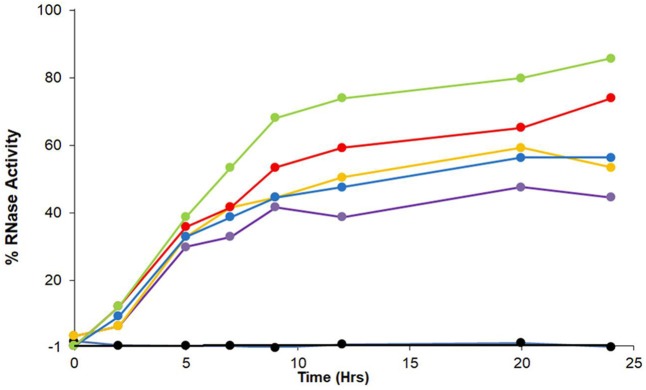
GSSG promotes refolding of reduced and denatured RNaseA. RNaseA was reduced and denatured as described in Figure 2. Refolding of reduced and denatured RNaseA was initiated by its dilution (200-fold) into renaturation buffer to a final concentration of 50 μg/mL. Refolding of RNaseA was either spontaneous and measured in the absence of oxidized glutathione, GSSG, (•), or in the presence of a mixture between GSSG (100 μM) and changing concentrations of reduced glutathione that ranged between 0 μM (•), 33 μM (•), 100 μM (•), 300 μM (•), and 1,000 μM (•). The percentage of refolding was calculated relative to native RNaseA activity. Similar results were obtained in two independent replicate experiments.

Refolding of rdRNaseA that took place in the presence of ZnJ2 and air, was rather slow. To examine whether ZnJ2 was also required under stronger oxidizing conditions, refolding of rdRNaseA was monitored in the presence of oxidized glutathione (GSSG). Figure 5 shows that rdRNaseA activity was rapidly restored in the presence of GSSG, even in the absence of the recombinant ZnJ2. Furthermore, the most efficient reactivation was observed in the presence of a mixture of GSH-GSSG, with gradually increasing concentrations of GSH. This could indicate that the mixture of reducing and oxidizing agents provided better conditions for the dynamic refolding, as compared to exclusively oxidizing conditions. However, when oxidation was slow, for example when air oxygen served as the oxidizing agent, oxidation and reactivation necessitated the presence of ZnJ2 (Figure 4). The conclusion from this series of experiments was that in itself ZnJ2 lacks oxidizing activity. However, both the wild-type and the thiol-deficient mutant possess a chaperone activity that is required for refolding of rdRNaseA in a mild oxidizing environment (air).

### ZnJ2 has no active refolding activity and does not contribute to active refolding activity of the DnaK/DnaJ/GrpE chaperone complex

**Figure 6:**
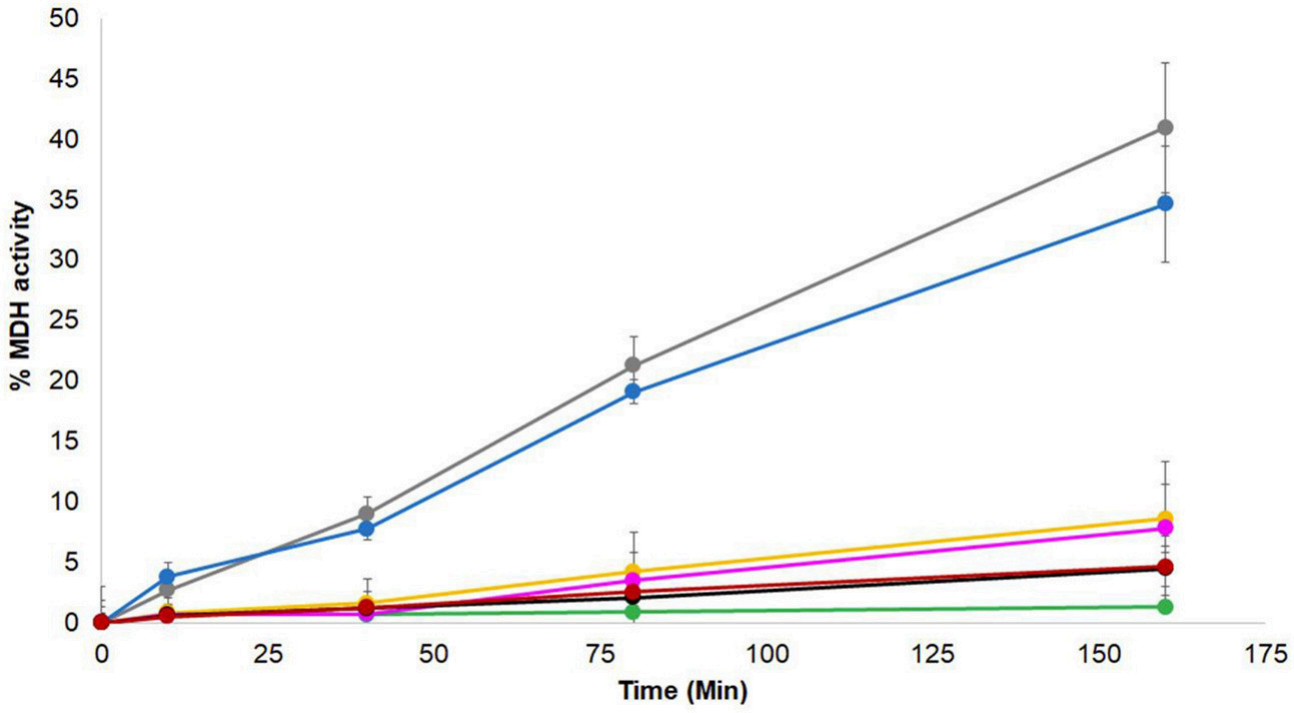
ZnJ2 does not interact with the KJE system in MDH refolding assay. Native MDH was heat denatured in 47°C for 30′ in the absence (•) or presence of different chaperone combinations. These included DnaK/ATP, GrpE, and DnaJ (•), DnaK/ATP, GrpE, and ZnJ2 (•), DnaK/ATP along with GrpE, DnaJ, and ZnJ2 (•), DnaK/ATP and GrpE (•), DnaJ alone (•), and ZnJ2 alone (•). Refolding in these tubes was performed at room temperature (25°C). GrpE was added during the refolding to assay tubes that contained DnaK/ATP. Refolding was monitored in the presence of pyruvate kinase and phosphoenol pyruvate. Aliquots were removed at various intervals (0, 10, 40, 80, 160 min) and MDH activity was monitored at 340 nm. The percentage of refolding was calculated relative to activity of native MDH. Similar results were obtained in two independent assays.

Genetic evidence from higher plants suggests that BSD2 is an RbcL-specific chaperone (Wostrikoff and Stern, 2007; Feiz et al., 2014). ZnJ2 from *C. reinhardtii* shows a sequence resemblance to the BSD2 from higher plants, but unlike the original BSD2, ZnJ2 comigrates with the *rbcL* transcript on translating polysomes, suggesting that it plays a key role in the biosynthesis of nascent RbcL chains (Doron et al., 2014). The chaperone capacity of ZnJ2 was formerly demonstrated by *in vitro* assays, showing that it could prevent the aggregation of denatured substrates, such as the citrate synthase, as well as its natural substrate, RbcL (recombinant). Thus, since ZnJ2 can prevent denatured proteins from aggregating, we examined whether it also possessed a refolding activity. This was done using a classical malate dehydrogenase (MDH) refolding assay, which was formerly used to monitor the ATP-dependent chaperone activity of the DnaK/DnaJ/GrpE system (Diamant and Goloubinoff, 1998). In this assay, MDH was heat-denatured at 47°C for 30 min in the presence or absence of different chaperones, until it lost all its activity, and then allowed to refold at 25°C, in the absence or the presence of different chaperone combinations. When the denaturation and renaturation were done in the presence of DnaK/DnaJ/GrpE chaperone system and ATP (Figure 6, gray circles), MDH was reactivated up to 35% of the native protein. Addition of ZnJ2 to the DnaK/DnaJ/GrpE mixture did not improve the refolding of MDH, and even slightly reduced it (blue circles), excluding the possibility of a synergism between ZnJ2 and the DnaK/DnaJ/GrpE system. Addition of ZnJ2 alone did not have by itself any refolding effect (black circles) and ZnJ2 could not replace DnaJ, when added to a mixture containing DnaK GrpE and ATP (yellow circles), indicating that its activity was not that of a DnaK co-chaperone. Lack of activity was also observed when MDH was denatured and renatured in the presence of DnaJ alone (red circles) or when refolding was spontaneous (green circles), as expected.

**Table:** 

MDH	–	ZnJ2	DnaK/ATP	DnaK/ATP,	DnaJ	DnaK/ATP/	DnaK/ATP
denaturation	–			ZnJ2		DnaJ	DnaJ, ZnJ2
Refolding	–	–	GrpE	GrpE	–	GrpE	GrpE
	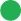	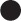	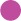	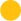	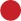	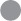	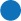

### ZnJ2 relates to the cysteine-rich domain of DnaJ type I

Several reports identified different novel proteins that contain a cysteine-rich center and are required for assembly of photosynthetic complexes, such as PSA2, a luminal protein required for photosystem I (PSI) biogenesis in *Arabidopsis thaliana* (Fristedt et al., 2014) and LQY1, a small thylakoid zinc finger chaperone with protein disulfide isomerase activity, which is involved in maintenance and repair of Photosystem II (PSII) in *A. thaliana* (Lu et al., 2011). Furthermore, the low similarity between the original maize BSD2 and its putative ortholog from *C. reinhardtii*, ZnJ2, and the different functions reported for these two proteins (Feiz et al., 2012; Doron et al., 2014), highlighted the possibility that there is a broad family of DnaJ-like proteins that have adopted specific substrates and functions that are associated with the rich repertoire of photosynthetic complexes in chloroplasts.

**Figure 7:**
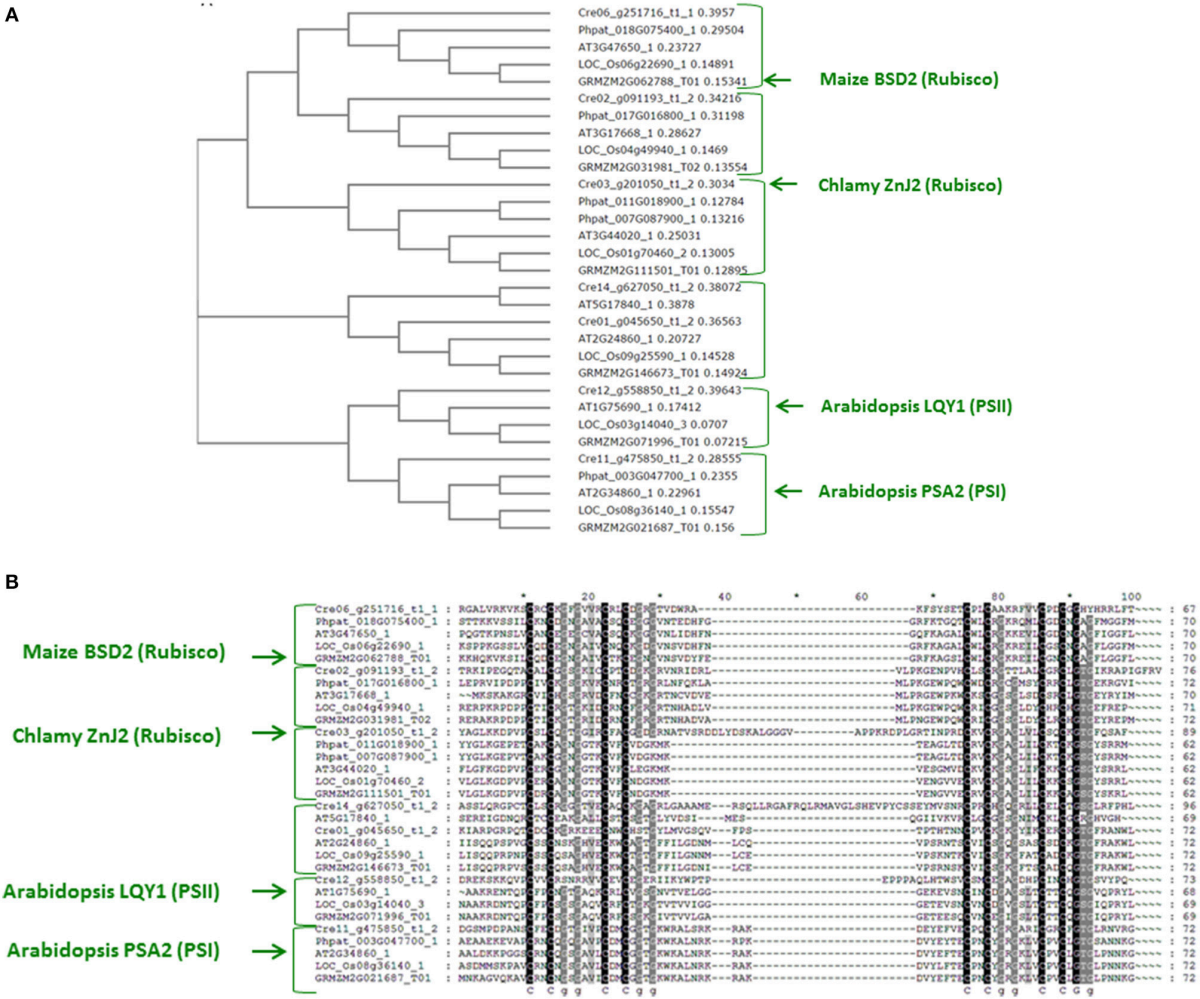
A family of ZnJ2-orthologs is found in photosynthetic organisms **(A)**. ZnJ2-orthologs were retrieved by a BLAST search in the Phytozome V.10 that was restricted to eukaryotic organisms. Searches were based on the amino acid sequence of ZnJ2 from *Chlamydomonas reinhardtii* (Cre03.g201050.t1.2) and the maize BSD2 (GRMZM2G062788_T01). The retrieved sequences were then aligned by Clustal Omega (former Clustal W2), and the multiple sequence alignment was used to generate a phylogenetic tree, based on the bootstrap test NJ (MUSCLE). The phylogenetic tree was based on genes derived from the genomes of *C. reinhardtii* (Cre03.g201050.t1.2, Cre12.g558850.t1.2, Cre06.g251716.t1.1, Cre11.g475850.t1.2, Cre01.g045650.t1.2, Cre02.g091193.t1.2, and Cre14.g627050.t1.2), *Z. maize* (GRMZM5G892742_T03, GRMZM2G111501_T01, GRMZM2G071996_T01, GRMZM2G062788_T01, GRMZM2G146673_T01, GRMZM2G031981_T02, and GRMZM2G021687_T01), *A. thaliana* (AT2G38000.1, AT3G44020.1, AT1G75690.1, AT3G47650.1, AT2G24860.1, AT3G17668.1, AT5G17840.1, and AT2G34860.1), *O. sativa* (LOC_Os01g70460.2, LOC_Os03g14040.3, LOC_Os06g22690.1, LOC_Os09g25590.1, LOC_Os08g36140.1, and LOC_Os04g49940.1) and *P. patens* (Phpat.016G067300.1, Phpat.011G018900.1, Phpat.018G075400.1, Phpat.003G047700.1, and Phpat.017G016800.1). **(B)**. The amino acid sequences of the regions that span the four cystein-rich centers (consisting of the partially conserved CXXCXGXG element) of the ZnJ2 orthologs were aligned by Clustal Omega. The alignment was further processed by GeneDoc version 2.7, to demonstrate sequence conservation. Conserved residues appear with a dark background that ranged from gray to black, based on the degree of similarity.

To address this possibility, the amino acids sequence of BSD2 from *maize* (GRMZM2G062788_T01) was used in a BLAST search of the proteome of *Chlamydomonas reinhardtii*, using the Phytozome database. The search was restricted mainly to enrich the output list with proteins that did not share additional domains with DnaJ type I family members, except for the Zn-finger, thus excluding closer orthologs of DnaJ proteins. The results of the search, shown in Figure 7A, revealed several Zn-finger domain proteins, Cre03.g201050.t1.2 (ZnJ2), Cre06.g251716.t1.2, Cre02.g091193.t1.2 and Cre12.g558850.t1.2 (the ortholog of AtLQY1). A parallel BLAST search with similar restrictions, using the amino acids sequence of the *C. reinhardtii* ZnJ2, generated four Zn-finger domain proteins, Cre02.g091193.t1.2, Cre14.g627050.t1.2, Cre11.g475850.t1.2 (also known as ZnJ1 and the ortholog of PSA2) and Cre01.g045650.t1.2, (also denoted as ZnJ3). Additional targeted BLAST highlighted the specific orthologs of each factor, among the genes that were identified in the initial searches based on the Zea maize BSD2 and the *Chlamydomonas* ZnJ2. A small database of orthologs was therefore generated from *Zea maize, Arabidopsis thaliana, Oriza sativa, Physcomitrella patens* and *Chlamydomonas reinhardtii*.

The Phylogenetic analysis of the proteins included in the database generated different clades, each containing one ortholog from the five different photosynthetic species (Figure 7B). The sequences were introduced into the ClustalW2 and a pairwise alignment was generated. A phylogenetic tree was generated by ClustalW2 using bootstrap testing for a neighbor-joining tree (Clustal W2_Phylogeny) (Saitou and Nei, 1987). The tree (Figure 7A) shows that Cre06.g251716.t1.2 is the most probable algal ortholog of the maize BSD2 (GRMZM2G062788_T01), while ZnJ2 (Cre03.g201050.t1.2) was found in a different clade with other orthologs from higher plants. This suggests that the algal ZnJ2 and the BSD2-ortholog belong to a novel family of cysteine-rich Zn-finger proteins that are responsible for heterologous roles in photosynthetic organisms. The conserved Zn finger domain of all the proteins is shown in Figure 7B, emphasizing the basis for their evolutionary conservation, as sharing a cysteine-rich domain similar to that DNAJAs.

## Discussion

ZnJ2 was formerly shown to be a chloroplast chaperone related to a small domain specific to the DNAJA subfamily but not to DNAJB&C (Doron et al., 2014). DnaJ is known to serve as a substrate targeting co-chaperone to the ATP-fueled unfoldase DnaK (Hsp70 in eukaryotes), alongside the nucleotide exchange factor GrpE (Martin and Hartl, 1997; Hartl and Hayer-Hartl, 2002). The resemblance between ZnJ2 and DnaJ Zn-finger domain led us to investigate the role of the cysteine-rich region in ZnJ2, using a variety of *in vitro* assays. Studies on the *E. coli* DnaJ has established that the highly conserved cysteine residues are involved in the coordination of two zinc ions per DnaJ monomer, which play a role in its chaperone activity (Banecki et al., 1996; Szabo et al., 1996; Linke et al., 2003). The ability of recombinant ZnJ2 to bind Zn atoms was demonstrated using the PAR/PCMB colorimetric assay. ZnJ2 was able to bind one Zn atom per molecule through its cysteine-rich domain. However, the role of Zn binding on its chaperone activity is still unclear. It has been shown that removal of the first zinc-binding domain of Ydj1 has no effect on the ability of the protein to suppress rhodanese aggregation (Lu and Cyr, 1998), whereas a similar truncation of *E. coli* DnaJ severely reduced its substrate binding activity (Szabo et al., 1996; Linke et al., 2003). However, under reducing conditions, the complete deletion of the Zn-binding domain from DnaJ did not significantly reduce the strict dependence of DnaK on DnaJ in order to actively refold heat-pre-aggregated G6PDH enzyme (Mattoo et al., 2014). In addition to its independent “holding” activity (Szabo et al., 1996), DnaJ was also shown to possess reductase and oxidase activities, but rather a weak isomerase activity (Tang and Wang, 2001). Since ZnJ2 shares the cysteine-rich functional element with a subdomain of bacterial DnaJ and of eukaryotic DNAJAs but not of bacterial CbpAs and eukaryotic DNAJBs, its chaperone activities were analyzed here, using biochemical assays that addressed different protein folding assisting functions.

We formerly showed that ZnJ2 can prevent the aggregation of both denatured citrate synthase and the denatured recombinant RbcL polypeptide following renaturation attempts. ZnJ2 could also hold on to thiol groups of insulin following their reduction with DTT and prevent aggregation of the beta chains (Doron et al., 2014). Here, we examined the additional chaperone activities of ZnJ2, trying to establish whether they are redox dependent. Reduced and denatured RNaseA has a natural ability to spontaneously refold to its native state *in vitro*. Native refolding occurs concomitantly with the formation of the correctly paired disulfide bonds under mild oxidizing conditions, and is driven by the thermodynamic stability of the native peptide configuration (Anfinsen et al., 1961). Using the RNaseA assay, we showed that ZnJ2 assists the refolding of the reduced and denatured enzyme, but this chaperone activity was redox-independent, since a mutant in which all cysteine residues were replaced had a similar activity. The refolding time for RNaseA was unusually long, as it gradually increased along 30–80 h, whereas canonical oxidoreductase activities usually peak within much shorter time periods. Thus, although the refolding and reactivation of RNaseA occurred only in the presence of ZnJ2, it required an oxidizing environment for the reformation of disulfide bridges, in addition to the chaperone activity. We found that air oxygen indeed slowly assisted the formation of the stabilizing S-S bonds, and that ZnJ2 was required to maintain the unstable refolding species poised to the optimal formation of these bonds. This explains why in the absence of ZnJ2, we observed no native refolding and why RNaseA activity was not resumed. However, the presence of ZnJ2 alone under anaerobic conditions was not sufficient to promote the reactivation of reduced denatured RNaseA, as it was not able to assist to the formation of the required disulfide bridges. Thus, ZnJ2 function was best exemplified when the RNaseA reactivation was slow in the presence of ~20% oxygen from the air. Furthermore, ZnJ2 was required only under conditions of slow oxidation, and was not required when oxidation occurred rapidly in the presence of GSSG. We also observed that the refolding effect was improved if allowed to occur in the presence of a mixture of reduced and oxidized glutathione, supporting the suggestion that the re-oxidation of disulfide bridges in the refolding protein is a dynamic process. The chaperone activity of ZnJ2 was further emphasized when compared to a control assay, in which its replacement with a non-chaperone protein control (citrate synthase), did not reactivate RNaseA, eliminating the possibility that specific chaperone activity of ZnJ2 is owed to a “crowding effect” of the biological environment (Dobson, 2003). In conclusion, although the RNaseA assay is usually employed to examine the oxidase activity of a protein (Pigiet and Schuster, 1986), here it highlighted that ZnJ2 has a thiol-independent chaperone activity and cannot promote oxidation of disulfide bridges.

The independent chaperone “holding” capacity of ZnJ2 was well established by different assays. We further examined if it could also cooperate with the ATP-dependent DnaK/DnaJ/GrpE chaperone system from *E. coli*, those close prokaryotic orthologues are also abundantly present in the chloroplast stroma, where RbcL is being synthesized. ZnJ2 is linked with nascent RbcL synthesis *in vivo*, as it comigrates with polysomes that are specifically loaded with the *rbcL* transcript. Since in bacteria and chloroplasts, synthesis of nascent polypeptides is accompanied and assisted by the prokaryotic DnaK/DnaJ/GrpE system, and since ZnJ2 shares an active Zn binding domain with DnaJ, the possibility that ZnJ2 cooperates with DnaK was examined *in vitro*, using the MDH refolding assay. Cooperation with DnaK in the MDH assay was observed also when small HSPs were included in the assay, as they stabilized the stress-denatured proteins for subsequent refolding by the multichaperone network (Veinger et al., 1998). However, a synergistic effect between ZnJ2 and DnaK was not observed, since addition of ZnJ2 to DnaK in the MDH refolding assay, with and without DnaJ, did not affect the MDH refolding and reactivation, as compared to that observed with DnaK/DnaJ/GrpE alone. Indeed, ZnJ2 does not contain a conserved J-domain with the characteristic HPD signature that can specifically dock into the nucleotide binding domain of ATP-bound DnaK and is the hallmark of bona fide J–domain co-chaperones. Therefore, a direct interaction between ZnJ2 and DnaK was not expected. However, ZnJ2 could have evolved to serve in *trans* a dedicated, yet complementary purpose during RbcL synthesis.

Recent reports highlighted a growing repertoire of DnaJ-like proteins that are required for the biosynthesis of specific complexes in the chloroplast. These could function at different stages of complex biogenesis, ranging from translation to assembly, and included proteins required for the accumulation of PSI and PSII complexes (Lu et al., 2011; Fristedt et al., 2014). The bioinformatic analysis indeed revealed a large number of proteins that share only the cysteine-rich center but do not contain any additional J-domain and protein binding domains, which are typical of DnaJ type I and DnaJ type II co-chaperones of DnaK. These were shown to cluster in specific clades, each representing a unique member that is encoded in each photosynthetic species. Interestingly, different members of this family appear to be involved in the separate stages of RuBisCO biogenesis, ranging from the synthesis of the RbcL polypeptide in the chloroplast (Doron et al., 2014), to the assembly of both RbcL and RbcS by CPN60/CPN20 (Gatenby and Ellis, 1990) in a process which is assisted by additional assembly factors, such as RAF1, RAF2, and BSD2 in eukaryotes and by RbcX in prokaryotes (Liu et al., 2010; Feiz et al., 2012, 2014). Our former report (Doron et al., 2014) showed that in addition to BSD2, RuBisCO biosynthesis in *Chlamydomonas* could be supported by ZnJ2. However, the relation between these two proteins was not resolved. Here we show that ZnJ2 is most probably not the direct ortholog of BSD2 in *C. reinhardtii*, although the two proteins belong to the same family of proteins. Our phylogenetic analysis reveals that another member of this protein family, namely Cre06_g251716_t1, shares the same clade with the maize BSD2, and not ZnJ2. Other members of this family were implicated in expression and accumulation of PSI (Fristedt et al., 2014) and PSII (Lu et al., 2011), emphasizing the requirement for a rich repertoire of substrate-specific chaperones during assembly of photosynthetic complexes in the chloroplast. Finally, the identification of the multiple of RuBisCO-specific chaperones, including BSD2, promoted its expression in *E. coli* (Aigner et al., 2017). Although ZnJ2 was not essential for expression of RuBisCO in a bacterial system, it could still have an important role during RuBisCO expression within the chloroplast.

## Author contributions

LD and MS conceived the study. PG contributed some proteins, plasmids, theoretical and technical advice. All authors contributed to the writing of the work and approved it for publication.

### Conflict of interest statement

The authors declare that the research was conducted in the absence of any commercial or financial relationships that could be construed as a potential conflict of interest.
